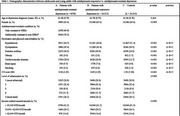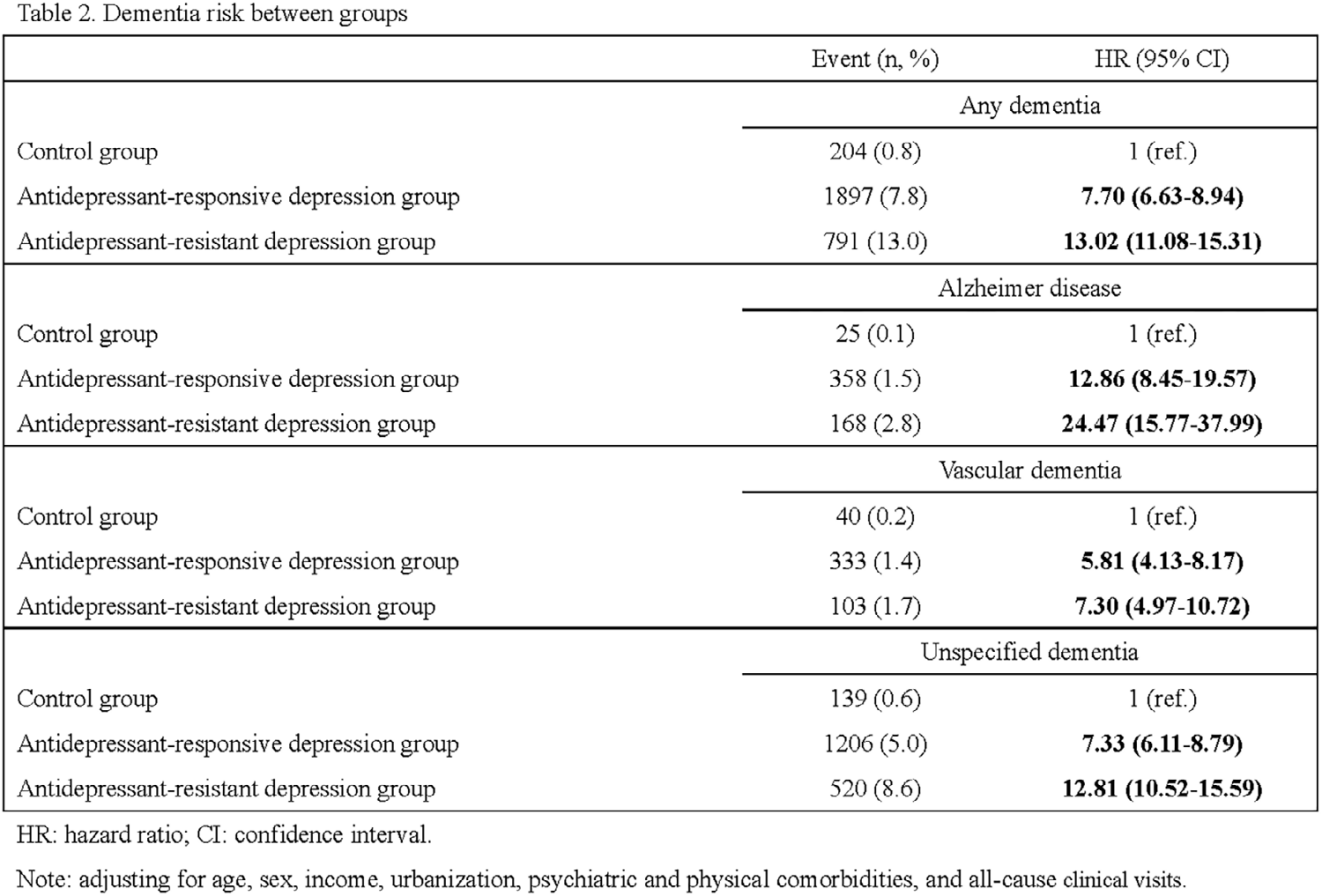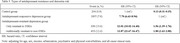# Antidepressant exposure and long‐term dementia risk in a nationwide retrospective study on elderly patients with major depressive disorder

**DOI:** 10.1002/alz70860_097934

**Published:** 2025-12-23

**Authors:** Che‐Sheng Chu, Chih‐Sung Liang, Mu‐Hong Chen

**Affiliations:** ^1^ Kaohsiung Veterans General Hospital, Kaohsiung, Kaohsiung, Taiwan; ^2^ Beitou Branch, Tri‐Service General Hospital, School of Medicine, National Defense Medical Center, Taipei, Taipei, Taiwan; ^3^ Taipei Veterans General Hospital, Taipei, Taipei, Taiwan

## Abstract

**Background:**

The use of antidepressants in elderly patients with major depressive disorder (MDD) was associated with elevated risk of dementia, but the results published are conflicting.

**Methods:**

Using the Taiwan Nationwide Health Insurance Research Database, 30,390 patients with MDD and 24,312 well‐matched controls were enrolled between 2002 and 2004. MDD patients were stratified into 6,078 patients with antidepressant‐resistant depression and 24,312 patients with antidepressant‐responsive depression, and all participants were followed up until the end of 2013. Those who developed any dementia, Alzheimer's disease (AD), vascular dementia (VaD), and unspecified dementia were identified.

**Results:**

Patients with antidepressant‐resistant depression and antidepressant‐responsive depression were more likely to develop any dementia, AD, VaD, and unspecified dementia than controls. Patients with antidepressant‐resistant depression group exhibited significantly higher risk of developing any dementia (hazard ratio [HR], 13.02 vs. HR, 7.70) and unspecified dementia (HR, 12.81 vs. HR, 7.33) than patients with antidepressant‐responsive depression, which was also higher than controls. Subsequent analysis demonstrated that patients with antidepressant‐resistant depression who were resistant to SSRIs only (HR, 12.01) or both SSRIs and non‐SSRIs (HR, 13.87) consistently showed higher risks of developing any dementia than patients with antidepressant‐responsive depression (HR, 7.70), which is also higher than controls. With antidepressant‐responsive depression group as reference group, patients with antidepressant‐resistant depression who were only resistant to SSRIs (HR, 1.56) and those with both resistant to SSRIs and non‐SSRIs (HR, 1.80) have higher risk of developing any dementia, but there was no significant difference between the two groups.

**Conclusions:**

The use of antidepressants in elderly patients with MDD was associated with elevated risk of dementia.